# Proceedings of the Physiological Temperance Society of the Medical Institute of Louisville

**Published:** 1842-04

**Authors:** 


Art. III,—*Proceedings of the Physiological Temperance Society of the Medical Institute of Louisville.* 1842, pp. 16.

This little pamphlet presents the proceedings of a Society which we announced in our January number. The time through which they run is two months, during which the Society obtained a charter from the State of Kentucky, had a certificate of membership lithographed, and a seal engraved; listened to four lectures, a report, and a valedictory, and acquired as members 137 out of the 263 pupils of the Institute. In the proceedings there are abstracts of the lectures, and a discourse, but we shall pass them by to insert the *Report.*

The Committee appointed to report how far the promotion of temperance should be regarded as a professional duty, offered the following, which was adopted and ordered to be published.

REPORT:

Extensive as may be the use of liquors rendered stimulating with alcohol, and intimately as they may be blended with our food, and the various innocent condiments, with which we stimulate ourselves, there is not, in the opinion of your Committee, the smallest necessity for their use as beverages. They impart no nourishment, and the excitement which they raise, is not only transient beyond that of many other stimulants, but followed by a degree of exhaustion, which more than counterbalances their exciting effects. Those effects, moreover, are chiefly found in the bodily desires and lower passions of the mind; and do not, therefore, prepare the individual for his duties, but, on the contrary, incapacitate him for them; which is equally true of labors that require strength of muscle, acuteness of bodily sensibility, soundness of judgment, or tranquillity of feeling.

Nor does the habitual use of alcoholic beverages ward off any form of disease. Your Committee might, but will not, insist, that there is *no* epidemic, in which these beverages would be preventive; but they fearlessly affirm, that if there be such, the individual who has been rigidly abstinent, up to the time when he begins their use for that purpose, will be far more likely to be benefited, than he who has habitually drunk them. To the strictly temperate, they would be medicines, and might, under proper circumstances, be preventives. There are, then, no physical reasons for the habitual use of alcoholic drinks.

Let us proceed to inquire whether there be physiological objections to their use; in other words, whether they occasion diseases.

1. An agent of such great activity as alcohol, cannot be used in large quantities, without injury to health. If the excessive use of the *supporters* of life, such as food, or warmth, or air with an over proportion of oxygen, be prejudicial to health, it will be admitted, that an agent which does not, in any manner or degree, contribute to nourish the body, and is still capable of exerting on it a powerful effect, must, if used in large quantities, prove a poison. Your Committee are willing to grant, that in smaller quantities, which they cannot undertake to define, alcohol may not disorder the functions. But they concur with universal experience, when they declare, that in the use of this, as well as other narcotic stimulants, there is a constantly increasing demand by the constitution; that this demand is much more importunate in some temperaments than others; that it is strictly physical; that in proportion as it increases, the will and moral powers of the individual are weakened; and, therefore, that the danger is imminent, that the person who begins with the temperate, will end with the intemperate use of whatever alcoholic beverage he may select. The diseases generated by this excess are various, according to the variety of drink, the age and temperament of the individual, his occupation, and the circumstances which surround him. Those which your Committee have most frequently observed in this country, are inflammation of the liver, followed by jaundice and dropsy; inflammation and ulceration of the mucous membrane of the stomach and intestines, with obstinate diarrhœa; vomiting, hawking and pyrosis in the morning, and in advanced cases, almost total loss of appetite; obstinate rheumatic inflammation, and occasionally gout; a mild bronchitis with cough, accompanied by a dirty, mucous expectoration, and an offensive breath; chronic inflammation of the eyes, with puriform discharge; nettle rash and leprosy; inflammation of the brain; epileptic convulsions; tremors and debility of the muscles; palsy of some of the muscles of the face, or one of the limbs, or of one side of the body; apoplexy; death.

Not wishing to present what might seem to be an exaggerated picture, your Committee forbear to extend this dark catalogue, leaving each member of the Society to add to it the results of his own future observation.

2. When none of the diseases, just enumerated, are developed, the abuse of intoxicating drinks very often creates a constitutional lesion or distemperature, which renders wounds and other injuries, and noxious impressions of various kinds, dangerous beyond what they would be under other circumstances. Thus a slight injury has sometimes led to uncontrollable and fatal inflammation and gangrene, in those who drank to excess; and no discreet surgeon would choose to perform an important operation on an individual who was habitually intemperate.

3. Intemperance multiplies diseases and shortens human life, by the exposure to which the intemperate are subject—1st, to mechanical accidents of various kinds—2nd, to the burning sun, producing *coup de soleil*—3rd, to severe cold, occasioning the loss of parts of their extremities, frequently of life itself—4th, to the neglect of personal cleanliness, causing a filthy and pedicular state of the skin—5th, to various vices, which become causes of disease. Thus drunkenness is the indirect cause of as many diseases as are directly produced by it.

But the ravages of intemperance are not limited to the body, for through it they are propagated to the mind. Sleeplessness, disordered hearing, seeing and feeling; reverie; delirium; monomania, and general madness, ending in fatuity, and leading to suicide or murder, are among the legitimate and melancholy effects. Alcoholic intemperance demands, then, the interference and aid of the medical profession, and, in the opinion of your committee, is not unworthy of deep and anxious attention. It would be superfluous for your committee to affirm, that the duty of laboring to prevent or avert diseases, is one of which no medical man can divest himself. But, still further, every profession, as an aggregate body, lies under some peculiar responsibilities of benevolence, and in its corporate capacity should prosecute them as public enterprises; and that assumed by our Society, your Committee regard as of this kind, and hope it will be so regarded by the profession at large. It only remains, then, to consider what can be done. 1. The members of the society, and of the profession generally, may accomplish a great deal by instruction, admonition and warning, put forth while mingling with the people in their daily exercises of duty. 2. They may exert themselves in practice, co-operating with those who benevolently labor to suppress intemperance, and, in fact exerting, if they choose, a more diffusive and salutary influence than any other class of men, because of their superior knowledge of the subject, and the confidence which the community at large repose in them on a subject so strictly professional. 2. They may sometimes make, with the consent, and even at the request of the intemperate, such administrations as will raise a disgust at alcoholic stimulants; they may supply excitements of a different kind to those who would seek substitutes; they may assure the deeply besotted, that they need not apprehend death from an instant discontinuance—and when such discontinuance is commenced, they may prescribe for the relief of the symptoms which arise from it. 5th, and lastly. They may refrain from drinking themselves, thus giving authority to their precepts and efficacy to their exhortation. Their salutary example would surpass, in its influence, that of any other class of men, while they would preserve themselves from intemperance, and redeem their professi on from the opprobrium under which it has rested since it first had an existence in the West.

Your Committee know, from observation and report, and are most happy to testify, that intemperance is much less prevalent in our profession than it was thirty years ago; and they believe that diminished drinking by physicians, is one of the causes of diminished drinking by the people; but there is both room and necessity for further reform on this point, and your Committee indulge the hope, that the present and still more the future labors of our Society, will be influential in its promotion.

DANIEL DRAKE,<br>

ALFRED MOORE,<br>

J. BERRIEN LINDSLEY, } Committee.<br>

ALFRED ADAMS,<br>

E. W. HORRELL,

We must also transcribe the following notices, commending them to the consideration of our readers.

### DONATIONS TO THE LIBRARY.

Having commenced the formation of a library of works on intemperance, the Society respectfully solicits of authors, publishers, and all other persons—books, lectures, discourses, orations, addresses and reports. It is desirous, also, to obtain publications on the effects of opium, tobacco, and other narcotic stimulants. Such as are usually transmitted by mail, may be forwarded through that channel, others by private hand as opportunity may offer. The whole to be directed—'Physiological Temperance Society of the Medical Institute of Lou-'sville, Kentucky."

### ORIGINAL CONTRIBUTIONS.

Intending to make a publication of its proceedings, annually, the Society is desirous of receiving original communications, of every kind, falling within its purview. Long as intemperance has prevailed in the world, the materials for its history are still imperfect, leaving ample opportunity for further observation and experiment. Our familiarity from childhood, with drunkenness, is one of the causes why our observations, as professional men, have not been so full and accurate as on many other forms of disease. The present wide spreading conviction, that alcoholic drinks are not only unnecessary but pernicious; in short, that their effects are morbid, will turn both the profession and the people to a closer study of them as diseased states; and thus many new facts will be brought to light. All communications of this kind should be directed to the Corresponding Secretary of the Society.

We trust that these requests will not be made in vain.

